# The Stroke and Carer Optimal Health Program (SCOHP) to enhance psychosocial health: study protocol for a randomized controlled trial

**DOI:** 10.1186/s13063-016-1559-y

**Published:** 2016-09-09

**Authors:** Catherine Brasier, Chantal F. Ski, David R. Thompson, Jan Cameron, Casey L. O’Brien, Nicola T. Lautenschlager, Graeme Gonzales, Ya-seng Arthur Hsueh, Gaye Moore, Simon R. Knowles, Susan L. Rossell, Rachel Haselden, David J. Castle

**Affiliations:** 1 Centre for the Heart and Mind, Australian Catholic University, Melbourne, VIC 3000 Australia; 2 Department of Psychiatry, University of Melbourne, Melbourne, VIC 3010 Australia; 3 Mental Health Service, St. Vincent’s Hospital, Melbourne, VIC 3065 Australia; 4 NorthWestern Mental Health, Melbourne Health, Melbourne, VIC 3052 Australia; 5 Department of Neurology, St. Vincent’s Hospital, Melbourne, VIC 3065 Australia; 6 Melbourne School of Population and Global Health, University of Melbourne, Melbourne, VIC 3010 Australia; 7 Department of Psychology, Swinburne University, Melbourne, VIC 3122 Australia

**Keywords:** Carer, Collaborative therapy, Cost-effectiveness, Dyad, Psychosocial, Randomised controlled trial, Stroke

## Abstract

**Background:**

Stroke is a leading cause of disability and distress, and often profoundly affects the quality of life of stroke survivors and their carers. With the support of carers, many stroke survivors are returning to live in the community despite the presence of disability and ongoing challenges. The sudden and catastrophic changes caused by stroke affects the mental, emotional and social health of both stroke survivors and carers. The aim of this study is to evaluate a Stroke and Carer Optimal Health Program (SCOHP) that adopts a person-centred approach and engages collaborative therapy to educate, support and improve the psychosocial health of stroke survivors and their carers.

**Methods:**

This study is a prospective randomised controlled trial. It will include a total of 168 stroke survivors and carers randomly allocated into an intervention group (SCOHP) or a control group (usual care). Participants randomised to the intervention group will receive nine (8 + 1 booster) sessions guided by a structured workbook. The primary outcome measures for stroke survivors and carers will be health-related quality of life (AQoL-6D and EQ-5D) and self-efficacy (GSE). Secondary outcome measures will include: anxiety and depression (HADS); coping (Brief COPE); work and social adjustment (WSAS); carer strain (MCSI); carer satisfaction (CASI); and treatment evaluation (TEI-SF and CEQ). Process evaluation and a health economic cost analysis will also be conducted.

**Discussion:**

We believe that this is an innovative intervention that engages the stroke survivor and carer and will be significant in improving the psychosocial health, increasing independence and reducing treatment-related costs in this vulnerable patient-carer dyad. In addition, we expect that the intervention will assist carers and stroke survivors to negotiate the complexity of health services across the trajectory of care and provide practical skills to improve self-management.

**Trial registration:**

ACTRN12615001046594. Registered on 7 October 2015.

## Background

Stroke is the second leading cause worldwide of death (11 %) and serious long-term disability [1, 2]. The significant burden of stroke extends across individuals, families and health systems globally [2, 3]. For the carer, a sudden shift from an acute hospital stay to informal care is experienced, as a family member or significant other contends with a new role and a dependent loved one [4, 5]. Equally important are the healthcare professionals who administer appropriate medical treatment and fulfil ongoing management and education roles for the stroke survivor across the illness trajectory [6]. However, in an oversubscribed and under-resourced health environment other innovative support methods are warranted. The Stroke and Carer Optimal Health Program (SCOHP) will adopt a person-centred approach combining collaborative therapy and care co-ordination to support and improve the mental and physical health of stroke survivors and their carers.

### Importance of the stroke-survivor-carer dyadic relationship

The strength of the dyadic relationship is crucial for achieving optimal mental and physical health for both the stroke survivor and carer. Alongside the stroke survivor, the carer must adjust to the immediate and long-term effects that require varying degrees of assistance and a consequent reduction in occupational and social activities [4, 7]. The nonprofessional carer role is complex and under-recognised encompassing information provision, managing emotions, social support, health maintenance and problem solving [8, 9]. The new-found role of carer is accompanied by intricacies and interdependencies including potential role reversals and unexpected physical, cognitive and emotional demands [8–11]. In addition, studies continue to report that early hospital discharge combined with a lack of appropriate planning can adversely impact rehabilitation and contribute to carer burden [12, 13]. Subsequently, carers also experience adverse health effects with high rates of depression, anxiety, increased morbidity and mortality [13–15]. This is of great concern given that informal carer involvement in rehabilitation is imperative to recovery.

### Stroke psychosocial interventions

In recent years research into the field of stroke has shifted from a physical emphasis to include psychological elements with a focus on carers; however, the stroke survivor/carer dyad has received minimal attention. Further, the evidence base regarding the effectiveness of support interventions for carers and/or stroke survivors is insufficient and inconsistent, primarily owing to methodological issues such as the diversity of intervention outcome measurements [6, 16, 17].

One of the most robust published studies was a randomised controlled trial of tailored psychoeducational modules and skill-building strategies (e.g. hands-on caregiver training and goal setting) delivered to 300 informal carers of stroke patients over three to five inpatient sessions and one home visit, which improved survivor and caregiver outcomes and reduced costs [18]. However, home visits are not always feasible and the individually tailored topics and goal setting focused more on the care of the stroke survivor than on the carer’s own self-care.

A recent critical analysis of 17 caregiver and 15 caregiver/stroke survivor dyad intervention studies produced evidence-based recommendations for the implementation and future design of stroke informal caregiver and dyad interventions [6]. Based on American Heart Association guidelines for classes and levels of evidence, interventions identified at the highest level of evidence were those that:

- combine skill-building (e.g. problem solving, stress management, goal setting) with psychoeducational strategies
- tailor interventions to the needs of stroke caregivers based on needs assessments along the continuum of care
- deliver the program face to face and/or by telephone (when in-person contact is not possible)
- offer an optimal number of sessions, which is between five and nine [6].

Unfortunately, few validated psychosocial interventions specific to carers are available, and for those that are, the mechanisms of effectiveness are rarely described [19]. A recent review evaluating the effectiveness of psychosocial interventions for informal carers found limited evidence regarding the effectiveness of psychosocial interventions, although psychoeducation, consisting of training in problem solving and stress coping, reduced depression and improved carer sense of competency at the trend level [16]. Overall, current limited evidence points towards more rigorous design of multidisciplinary psychosocial interventions, sustainability of outcomes and inclusion of the stroke survivor-carer dyad.

### Translating Research, Integrated Public Health Outcomes and Delivery (TRIPOD)

This randomised controlled trial (RCT) is part of a larger research program – TRIPOD – which will evaluate our Optimal Health Program (OHP) across three chronic conditions; namely stroke, diabetes mellitus and chronic kidney disease, including cost-effectiveness analyses. Based on a collaborative therapy framework [20], the OHP was originally developed to support people with mental illness [21, 22]. The initial trial, in an adult mental health service, demonstrated significant improvements in health and social functioning, reduced hospital admissions and net cost savings per patient [22]. A key aspect of collaborative therapy is recognising that ‘recovery’ and chronic models of health care are not dichotomous [20]. With the intention of enhancing self-efficacy, self-management, care co-ordination and quality of life, the OHP has been adapted within the broader context of chronic disease. Thus, in the current series of trials our OHP is used to implement this therapeutic framework to enable clinicians and consumers to work systematically towards the achievement of optimal psychosocial health outcomes within mainstream health services [23]. The self-management foundations of the OHP are particularly relevant for adults affected by stroke and their carers who face the daily challenge of managing various and often simultaneous aspects of their disease such as managing multiple medications, cognitive training, ongoing appointments, and physiotherapy as well as coping with the emotional impact of stroke and their care regimen. This protocol describes an RCT (SCOHP) that has been designed to evaluate the OHP for those affected by stroke – survivors and carers.

### Qualitative study: informing development of an optimal health program

Healthcare provider experiences of carers have been researched, but little is written about how these can inform development of support programs. In collaboration with the National Stroke Foundation, Carers Victoria and three consumers (one carer and two stroke survivors) a qualitative study was undertaken to inform development of an Optimal Health Program (OHP) to support carers of those who have experienced a stroke [24]. The aims of the qualitative study were to inform SCOHP by: (1) exploring healthcare provider perceptions of stroke carer roles and support needs and (2) examining carer needs across the stroke care trajectory. To achieve this, we conducted four semi-structured focus groups (*n* = 23) of stroke healthcare providers across acute, subacute, and community rehabilitation services. Focus group facilitators used a semi-structured focus group schedule to guide discussions. Sessions were then recorded, transcribed, and analysed using thematic and content analysis. Table 1 shows the three key themes and sub-themes that emerged from the data, which highlight the distinct roles of healthcare providers and carers.

**Table 1:** Themes and sub-themes from thematic analysis

Themes	Sub-themes
Transition	Healthcare provider roles across stages of the stroke trajectory
	Carer transition to a caring role and how this changes over time
Information	Delivery of information by healthcare provider
	The carers’ response to information and difficulties comprehending implications
Impact of stroke	Healthcare provider role in supporting the carer and person with stroke and maintaining hope
	Carers’ experiences of the impact of stroke

**Table 2:** Primary and secondary outcome assessments and time points for SCOHP

	Carer				Stroke survivor			
Assessment tools	BL	3	6	12	BL	3	6	12
Primary outcomes								
AQoL-6D (20 items)	X	X	X	X	X	X	X	X
GSE (10 items)	X	X	X	X	X	X	X	X
Secondary outcomes								
BIPQ (8 items)					X	X	X	X
Brief COPE (28 items)	X	X	X	X	X	X	X	X
CASI (30 items)	X	X	X	X				
CEQ (6 items)	X				X			
EQ-5D -3 L (6 items)	X	X	X	X	X	X	X	X
HADS (14 items)	X	X	X	X	X	X	X	X
HCUQ (10 items)	X	X	X	X	X	X	X	X
MCSI (13 items)	X	X	X	X				
TEI-SF (9 items)				X				X
BFI-10 (10 items)		X				X		
WSAS (5 items)	X	X	X	X	X	X	X	X

*AQoL*-*6D* Assessment of Quality of Life-6 dimensions, *GSE* General Self-Efficacy Scale, *BIPQ* Brief Illness Perceptions Questionnaire, *Brief COPE* abbreviated version of the COPE Inventory, *CASI* Carers’ Assessment of Satisfaction Index, *CEQ* Credibility/Expectancy Questionnaire, *EQ*-*5D*-*3L* European Quality of Life-5-dimensions-3 levels, *HADS* Hospital Anxiety and Depression Scale, *HCUQ* Health Care Utilisation Questionnaire, *MCSI* Modified Caregiver Strain Index, *TEI*-*SF* Treatment Evaluation Inventory-Short Form, *BFI*-*10* Big Five Inventory-10 item, *WSAS* Work and Social Adjustment Scale

The findings of this study were used to inform the development of the OHP, specifically in terms of having: staged information across the illness trajectory; flexible support during transition periods; and a balance of practical tools and empathic communications around the impact of stroke. In summary, the discussions held with health providers supported the integration of an OHP for carers within existing stroke care services across acute and community settings.

### Research aims

The aim of the study is to determine whether a stroke-specific OHP (SCOHP) improves the psychosocial health of stroke survivors and their carers, compared to usual care. The primary objective is to identify the impact of the OHP on levels of self-efficacy and quality of life for those affected by stroke. Secondary objectives are to evaluate the impact of the SCOHP on depression, anxiety, social and workplace functioning, self-management, and illness perceptions of and coping with stroke, and carer strain and satisfaction.

In addition, a health economic cost analysis will be performed, assuming an Australia-wide implementation, to identify any cost savings of SCOHP over current practice. Quality-adjusted life years (QALYs) will be measured using the Assessment of Quality of Life-6D (AQoL-6D) [25] and European Quality of Life-5 dimensions-3 levels (EQ-5D-3L) [26]. Process evaluation using focus groups will also be conducted with patients and clinicians to assess the effectiveness of the SCOHP, implementation, uptake and service delivery.

## Methods

### General design

This is a prospective randomised controlled trial to evaluate the effectiveness of the SCOHP for improving the psychosocial health of those who have experienced stroke and their carers. The SCOHP will be delivered as an 8-week individualised support program, with an additional booster session, and will be compared to usual care. Assessments will take place at baseline, 3, 6, and 12 months. The study protocol was approved by the St Vincent’s Hospital Human Research Ethics Committee (HREC-A 019/14). An executive steering committee (all authors) oversees project planning, conduct and ongoing data collation.

### Setting

The study will be conducted at the neurology unit of St Vincent’s Hospital, a large metropolitan teaching hospital in Melbourne, Australia. Between 2011 and 2012, 737 patients were admitted to St Vincent’s Hospital, with a principal diagnosis of stroke. The stroke unit at St Vincent’s Hospital, Melbourne will enable planned recruitment of 168 participants for the SCOHP program over a 2-year period.

### Participants

A total of 84 patients diagnosed with stroke, and 84 carers of these patients, will be recruited into the RCT. For the purposes of this study, stroke is defined as cerebral infarction or parenchymal haemorrhage confirmed by medical records. The following criteria are to be met for inclusion into the RCT: (1) diagnosis of stroke for patient or self-nominated carer of a stroke patient; (2) 18 years or older; (3) ability to converse in English without an interpreter or professional assistance; (4) absence of developmental disability or amnestic syndrome impairing their ability to learn from the intervention; and (5) absence of serious comorbid illness, including severe forms of aphasia, as identified by the nurse unit manager, and cognitive impairment, as identified from medical notes scoring lower than 24 on the Mini-Mental State Examination (MMSE) [27]. As the OHP adopts a holistic approach to managing chronic disease, patients may enter the program at any stage along the continuum of care.

Power was calculated to detect a medium effect size of Cohen’s *d* = 0.50. This was chosen as a clinically meaningful effect size that may be compared with previous RCT research in the area of chronic disease management programs [28]. Calculations assumed two primary outcomes (health-related quality of life and General Self-Efficacy Scale (GSE) scores), four assessment points (baseline, 3-month, 6-month, and 12-month), a study-wide type I error rate (α) of .05, and hence a type II error rate (β) of 0.20 (power of 0.80), a correlation of post-treatment scores with baseline measurements (ρ) of 0.81, and a two-tailed statistical test [29]. To detect an effect size of Cohen’s *d* = 0.50, 53 participants in each of the control and intervention groups will be required. Allowing for up to 20 % attrition, a total of 168 participants, or 42 carers and stroke survivors in control and intervention groups will be recruited.

### Study procedures

#### Recruitment

**Fig. 1:**
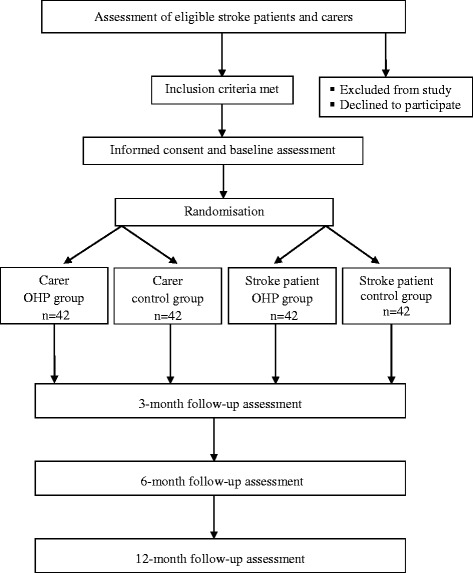
Flowchart of the Stroke and Carer Optimal Health Program (SCOHP) randomised controlled trial (RCT)

Potential patients who have been diagnosed with stroke and/or their carer will be identified by clinical staff (e.g. neurologist, nurse) and provided with a study flyer. Patients and/or carers will be asked permission for a researcher to approach them to discuss the program in more detail. If agreeable, they will be approached, informed and formally consented by the research assistant. Study fliers will also be posted online through community organisations and will include contact details for the research team. Participants from the community may contact researchers directly to request further information. Planned recruitment will occur over an 18-month period (see Fig. 1).

#### Consent

The process of consent will be in accordance with the Declaration of Helsinki. Nurse unit managers were consulted to determine a patient’s eligibility for the study. Senior clinicians and the research team were consulted in instances when it was unclear if an individual met the inclusion criteria. All eligible patients and carers will be fully informed that they are being asked to participate in an RCT. The procedures involved in the study, and the chances of being assigned randomly to one of two groups will be explained verbally and via an information sheet approved by the hospital’s Human Research Ethics Committee. A signed consent form will be obtained from each participant. Participants will be made aware of their right to withdraw from the study at any time without any effects on their clinical management.

#### Randomisation and blinding

Using a computer-generated block randomisation sequence created by a researcher independent of the study, participants will be allocated to treatment or control group. The allocation sequence will be generated using random numbers and participants will be randomised progressively as they consent. Patients and carers will be randomized as dyads. Patients or carer will be randomized alone if they are not participating as a dyad. Due to the nature and length of the intervention, it is not possible to blind either participant or investigator to the treatment allocation.

#### Intervention: SCOHP

The SCOHP is delivered at a nominated place of convenience by the participant i.e. home, hospital, community health centre. Dyads have the option of either receiving the intervention independently or together. The SCOHP comprises a modular format of eight sequential sessions plus a booster, based on a structured workbook. Participants are encouraged throughout the program to identify areas of stroke- or carer-related health concerns on which they would like to focus. Sessions are approximately 1 hour in duration and held weekly, apart from the ‘booster’ session, which is held 3 months after session 8. Learning is cumulative with each session designed to build on the previous session including tasks to complete between sessions, i.e. journaling and coping strategies (e.g. breathing exercises).

In summary, session 1 introduces SCOHP within the six domains of the ‘Optimal Health Wheel’: social, physical, emotional, intellectual, employment and spiritual as documented in the workbook. This session provides participants with the opportunity to explore and understand stroke self-management behaviour from a holistic perspective. Sessions 2 and 3 initiate development of a health plan exploring the implications and potential complications of stroke in terms of strengths and vulnerabilities, and understanding and monitoring disease impact (e.g. emotional burden and physical weakness). Session 4 focuses on medication management and metabolic monitoring. Session 5 expands the health plan to include key stroke partnerships and supports in the community and online (e.g. www.strokefoundation.com.au). Change enhancement is the focus in session 6, in terms of understanding past events and establishing new proactive avenues for change. The aim of session 7 is goal setting via creative problem solving and planning around the complexities of stroke. To cement a shift in focus of the person’s illness from being ‘dependent on’ services to being ‘supported by’ services, session 8 strategises stroke advanced care planning that incorporates wellbeing maintenance and sustainability. The goal of the ‘booster session’ (session 9) is to review health plans, consolidate progress, and reflect on achievements towards health-related goals.

A health professional (e.g. nurse, psychologist) trained in the approach (2-day workshop plus regular supervision and fidelity checks) will facilitate each session. The facilitator will draw on carer and stroke-specific information in concordance with individual circumstances. Examples include the relationship between depression and caregiving or physical impairments of stroke, availability of stroke and carer supports in the community, and coping strategies for addressing anxiety and stress related to new roles and circumstances. The emphasis is on collaboration between facilitator and participant to arrive at goals for the program that stem from the participant’s main concerns and needs. The facilitator will encourage participants to identify their early warning signs of stress and illness and integrate healthy coping strategies to prevent the build-up of stress. Facilitators may also discuss and arrange referrals for other services in conjunction with the multidisciplinary team depending on participant needs. Additionally, facilitators will work with the multidisciplinary team to coordinate visits. Participants in rural and regional areas will have the option of participating in sessions via phone or Skype.

#### Control

The comparison group will receive usual care and no SCOHP intervention. As participants will be recruited from a variety of settings (hospital outpatients, community organisations) we anticipate variation in standard care received. To capture this variation, all participants will complete the Health Care Utilisation Questionnaire (HCUQ) [30] at each time point. Participants in the control group will have the option of completing the SCOHP at the end of the trial once evaluation is complete.

#### Outcome measurements

Table 2 details the primary and secondary outcome measures and time points for carers and stroke survivors. Participants complete the measures independently unless a specific request is made for assistance e.g. due to vision or motor skill impairment. Primary outcome measures for both stroke survivors and carers are quality of life and self-efficacy. Health-related quality of life will be assessed using the (AQoL-6D) [25], which consists of six dimensions of health and a global ‘utility’ score and the EuroQol-5D (EQ-5D) [26]. Self-efficacy is to be assessed using the General Self-Efficacy Scale (GSE) [31] a measure of perceived self-efficacy in response to daily challenges and stressful life events. Secondary measures for both stroke survivors and carers are: coping strategies as measured using an abbreviated version of the COPE inventory, the Brief COPE [32]; symptom severity and caseness of depression and anxiety disorders as assessed using the Hospital Anxiety and Depression Scale (HADS) [33]; a 10-item measure of the Big Five personality dimensions (BFI-10) [34]; effect of an individual’s mental health on their ability to function via the Work and Social Adjustment Scale (WSAS) [35]; treatment expectancy and rationale credibility of the clinical study as assessed with the Credibility/Expectancy Questionnaire (CEQ) [36]; perceived satisfactoriness of treatment as assessed using the Treatment Evaluation Inventory-Short Form (TEI-SF) [37]; and health care utilisation and its economic impact assessed by the Health Care Utilisation Questionnaire (HCUQ) [30]. Stroke survivors will also be assessed for cognitive and emotional responses to stroke using the Brief Illness Perceptions Questionnaire (BIPQ) [38]. In addition, carers will be assessed for carer strain using the Modified Caregiver Strain Index (MCSI) [39] and carer satisfaction as assessed by the Carer Assessment of Satisfaction Index (CASI) [40].

Due to the potential for variability of ‘usual care’ in the control group, key aspects of usual care will be assessed with the HCUQ. Furthermore, medical records will be reviewed to determine stroke diagnostic information and clinical indices including the Modified Rankin Scale (MRS), which measures the degree of disability/dependence after a stroke.

#### Program assessment and treatment fidelity

The SCOHP facilitators will be trained in program delivery, receive a structured manual/protocol and monthly group supervision with the clinical investigators (with individual supervision provided as needed in between group sessions). The purpose of supervision will be to discuss problems in study procedures and ensure standardised activity. The SCOHP sessions will be audio recorded with a random selection rated by independent assessors in compliance with the SCOHP protocol. Variations from the protocol will be identified and relayed to the facilitator. Facilitators will complete a summary of each session using a standard template and send these notes to the research team. Session notes will include OHP topics covered, participant concerns raised, and needs for supervision. Additionally, content of sessions regarding participant requirement and concerns will be discussed at supervision meetings.

Post-intervention focus groups will be held for clinicians and participants. Participants will be informed during consent (both written and verbal) of the option to participate in focus groups, and that the purpose is to ascertain an in-depth understanding of their experiences of the study, advantages and disadvantages of conducting the study/program in their services (for clinicians), and recommendations for components to include or exclude from the SCOHP. It will be made clear to participants when consenting that the number of focus groups will be limited; such that they will only be run until data saturation is achieved. It is envisioned that data saturation will be reached after 2 to 3 focus groups, each containing 8 to 12 individuals. To increase objectivity, focus group facilitators will be independent researchers who were not OHP facilitators. The pragmatic data analysis approach of Halcomb and Davidson [42] will be used for the purpose of focus group data analysis. In summary, identifying key passages and words will be independently analyzed, coded, and categorized (classifying key passages and words within themes) drawing on pragmatic thematic analysis to form emergent themes.

#### Statistical analyses

Intention-to-treat analyses will be employed to prevent overestimation of efficacy. Categorical variables will be analysed using chi-squared tests (or Fisher’s exact test for small samples). A mixed-effects model, repeated measures (MMRM) approach will be used to examine the longitudinal profile of continuous variables at 3, 6 and 12 months post-baseline. For all MMRM analyses, baseline scores will be used as covariates and the models will include prespecified fixed effects of treatment, clinician, and time, and treatment-by-time and treatment-by-clinician interactions.

Secondary analyses using analysis of covariance will be conducted to compare change scores during treatment and follow-up phases for primary, secondary, and process outcomes using the fixed, continuous covariate of baseline score as well as the categorical fixed effects of treatment group, clinician, and treatment-by-clinician interactions.

Although the attrition rate is not expected to vary by treatment condition, we will attempt to identify key predictors of attrition status (i.e. demographic and baseline clinical characteristics) and test for differences between conditions. Assuming the data are missing at random, several procedures offer effective approaches that may attenuate attrition. Maximum likelihood models (i.e. MMRM), with time as a random variable, allow the use of all available data from all assessments, reducing bias and increasing power [43]. In addition, multiple imputation procedures that utilise the expectation-maximization (EM) algorithm with bootstrap estimates of standard errors will be used to address attrition. The application of these procedures can provide unbiased estimates, even in the face of substantial missing data [44].

A full economic evaluation will occur alongside the proposed RCT. Healthcare outcomes and costs will be compared between participants in the control and interventional conditions. Healthcare system (medical record) and self-reported information via the HCUQ [30] will be used to generate analyses. The utility measurements of participant quality of life will be assessed using AQoL-6D [25] developed in Australia and the EQ-5D-3L [26] developed in Europe. The potential long-term (lifetime) impact on cost and effectiveness of intervention beyond the trial period will be extrapolated using the Markov process modelling method.

## Discussion

Stroke can carry severe consequences for the patient and their informal carers or family members who often feel inadequately prepared to deal with the physical, cognitive and emotional demands [1–3]. Carers experience adverse health effects with high rates of depression [13], anxiety [14] and mortality [15]. The informal caring role is pivotal in maintaining stroke survivors in the community but this comes at a significant cost to the carer [4, 9–12]. It is therefore important to develop programs that will support the carer’s coping and minimise the level of burden and ill-health they experience.

The crucial evidence gap lies in the integration and co-ordination of patient and carer support programs within health service delivery. Integral to SCOHP is its integration of carer and patient support within health services from acute to community care. Engaging with multiple clinicians can be a daunting task, both for patients and informal carers. The SCOHP assists in negotiating this complexity by adopting a person-centred approach across the patient trajectory. In addition, stroke survivor and stroke carer psychosocial health is rarely studied as a dyad, thus this RCT is expected to make a significant contribution to improve the mental health and wellbeing of patients who have experienced stroke and their carers.

There are several strengths to this study protocol. Primarily, in the inclusion of the ‘patient-carer dyad’ tailored to each individual, for both intervention and assessment purposes. Integration and rollout of the RCT in a clinical setting was purposefully incorporated to identify the adaptability of the intervention to a ‘real-world setting’, i.e. co-ordination and communication between departments. If successful, the simultaneous evaluation of RCTs across three of the most burdensome chronic conditions will provide evidence for the potential applicability of the intervention to extend to other chronic diseases. To our knowledge this is the first trial to include a comprehensive health economic cost analysis in the assessment of an educational, psychosocial intervention aimed at improving the mental and physical health of stroke survivors and their carers.

This series of trials follows common ethical principles applied in RCTs. Participants receive verbal and written information before consenting and before study procedures, they are not exposed to any risks, participation is voluntary and they may withdraw at any time without reason and without their usual care being affected in any way. Participants in the control group are also offered the intervention at the end of the follow-up period.

### Trial status

Patient recruitment was ongoing at the time of manuscript submission. Data collection will continue until at least December 2017.

## Abbreviations

AQoL-6D: Assessment of Quality of Life-6 dimensions
BFI-10: Big Five Inventory-10 item
BIPQ: Brief Illness Perceptions Questionnaire
Brief COPE: abbreviated version of the COPE Inventory
CASI: Carers Assessment of Satisfaction Index
CEQ: Credibility/Expectancy Questionnaire
EM: expectation-maximization
EQ-5D-3L: European Quality of Life-5 dimensions-3 levels
GSE: General Self-Efficacy Scale
HADS: Hospital Anxiety and Depression Scale
HCUQ: Health Care Utilisation Questionnaire
MCSI: Modified Caregiver Strain Index
MMRM: Mixed-effects Model, Repeated Measures
MMSE: Mini-Mental State Examination
MRS: Modified Rankin Scale
OHP: Optimal Health Program
QALY: quality-adjusted life year
RCT: randomised controlled trial
SCOHP: Stroke and Carer Optimal Health Program
TEI-SF: Treatment Evaluation Inventory-Short Form
TRIPOD: Translating Research, Integrated Public Health Outcomes and Delivery
WSAS: Work and Social Adjustment Scale
